# Halometabolites isolated from the marine-derived fungi with potent pharmacological activities

**DOI:** 10.3389/fmicb.2022.1038487

**Published:** 2022-10-04

**Authors:** Yu Chen, Lian-Cheng Xu, Shan Liu, Zi-Xiang Zhang, Guan-Yi Cao

**Affiliations:** ^1^Department of General Surgery, Suqian First Hospital, Suqian, China; ^2^Department of General Surgery, The First Affiliated Hospital of Soochow University, Suzhou, China

**Keywords:** halometabolites, natural products, marine fungi, chemical diversity, biological activities

## Abstract

Halometabolites, usually produced in marine environment, are an important group of natural halogenated compounds with rich biological functionality and drugability and thus play a crucial role in pharmaceutical and/or agricultural applications. In the exploration of novel halometabolites from marine microorganisms, the growing number of halogenated compounds makes it necessary to fully present these metabolites with diverse structures and considerable bioactivities. This review particularly focuses on the chemodiversity and bioactivities of halometabolites from marine-derived fungi. As a result, a total of 145 naturally halogenated compounds, including 118 chlorinated, 23 brominated, and four iodinated compounds, were isolated from 17 genera of marine-derived fungi. Interestingly, many of halometabolites, especially for the brominated and iodinated compounds, are generated by the substitution of bromide and iodide ions for the chloride ion in cultivation process. In addition, these compounds possess diverse structural types, which are classified into polyketides (62.7%), phenols (16.6%), alkaloids (14.5%), and terpenoids (6.2%). Their cytotoxic, antibacterial, and anti-inflammatory activities indicate the high potential of these halogenated compounds as lead compounds for drug discovery.

## Introduction

Halometabolites are a group of natural halogen-containing (Cl, Br, I, F) compounds which possess rich biological functionality and drugability. It is estimated that more than 5,000 halogenated compounds have been reported (Liao et al., 2016). Among them, chlorination is the predominant occurance, and then followed by bromination, while iodination and fluorination are extremely rare (Neumann et al., 2008). Halometabolites are generally produced from abiogenic and biogenic pathways. Biogenic halometabolites are formed by microorganisms (fungi and bacteria), plants, algae, and marine invertebrates (sponges and corals) (Kasanah and Triyanto, 2019). Biosynthetically, enzymatic halogenation through halogenases such as flavin adenine dinucleotide-dependent halogenases (FDHs) and non-heme Fe^II^/α-ketoglutarate halogenases is the most common way to these compounds (Neumann et al., 2008; Liao et al., 2016). Halometabolites possess high diversity in structure, ranging in complexity from simple halogenated indoles, terpenes, and phenols to miscellaneous polypeptides and polyketides.

Apart from their novel structures, the presence of halogens in natural products significantly enhances their biological activities. The halogen substituents are responsible for the bioactivity, bioavailability, and stability of the compounds (Kasanah and Triyanto, 2019). Halometabolites also play an important role in pharmaceutical and agricultural applications. Many of them have been used for decades as pharmaceuticals and agrochemicals. It is worth mentioning that natural products have benefited significantly from the growth of the pharmaceutical industry, especially of pharmacologically attractive lead drugs and potential clinical therapeutic drugs. Among them, approximately 25% of clinically therapeutic drugs are halogenated, indicating halogen substituents as remarkable contributors to pharmacological applications. A large number of halogenated natural products-inspired pharmaceuticals are either FDA or EMEA approved. Representative examples of them include the antibiotics chloramphenicol and vancomycin, the anticancer drugs salinosporamide A, spongistatin, rebeccamycin, and calicheamicin (Supplementary Figure S1; Niu et al., 2021). Therefore, in this sense, halometabolites bioprospecting is a considerable approach to discover new innovative drugs.

Compared to those from terrestrial plants, halometabolites derived from marine environment are relatively unexplored. The marine environment is a crucial source of halotolerant microorganisms (Wang et al., 2011). Microorganisms living in marine extreme environment are suffered from low temperature, high pressure, high salinity, and low oxygen concentration, and have evolved extraordinary metabolic pathways to produce novel secondary metabolites (Xu et al., 2020). Marine-derived fungi have been largely explored due to their ability to generate structurally novel secondary metabolites with remarkable biological activities. Given the crucial role that halogen substituents can play in the bioactivity of these metabolites, high metabolic potential of halometabolites production can be expected from the marine-derived fungi. This present review illustrates the chemistry and biological activities of halometabolites produced by marine-derived fungi. A total of 145 naturally halogenated compounds, including 118 chlorinated, 23 brominated, and four iodinated compounds, were isolated in the past decades. Crucial insights into their chemical diversity and biological activities are provided herein. This review will reveal these halogenated compounds as lead compounds for the development of innovative drugs.

## Chemical diversity and biological activity

### Halogenated polyketides from marine-derived fungi

#### Azaphilones

**Figure 1:**
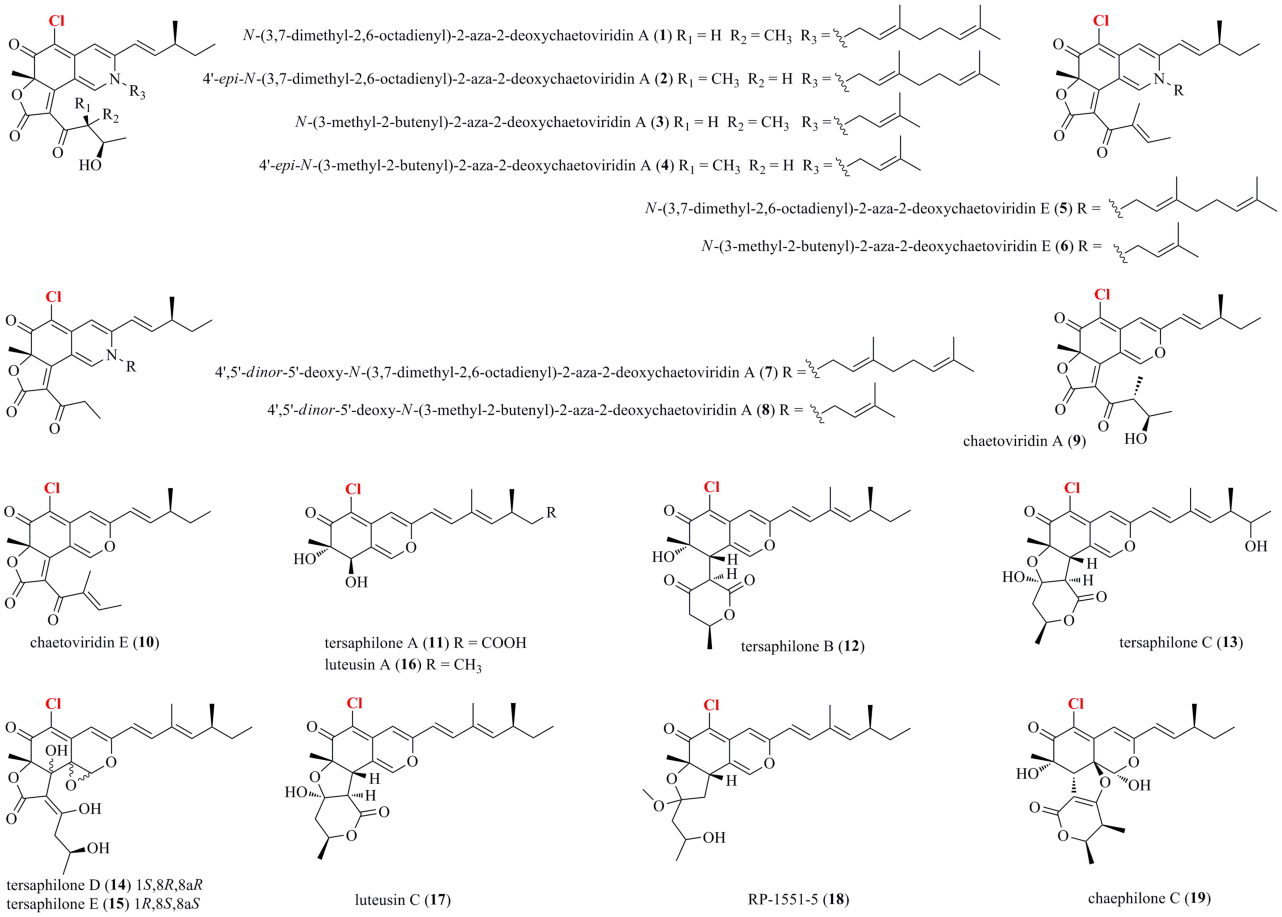
Halogenated azaphilones from marine-derived fungi (**1–19**).

**Figure 2:**
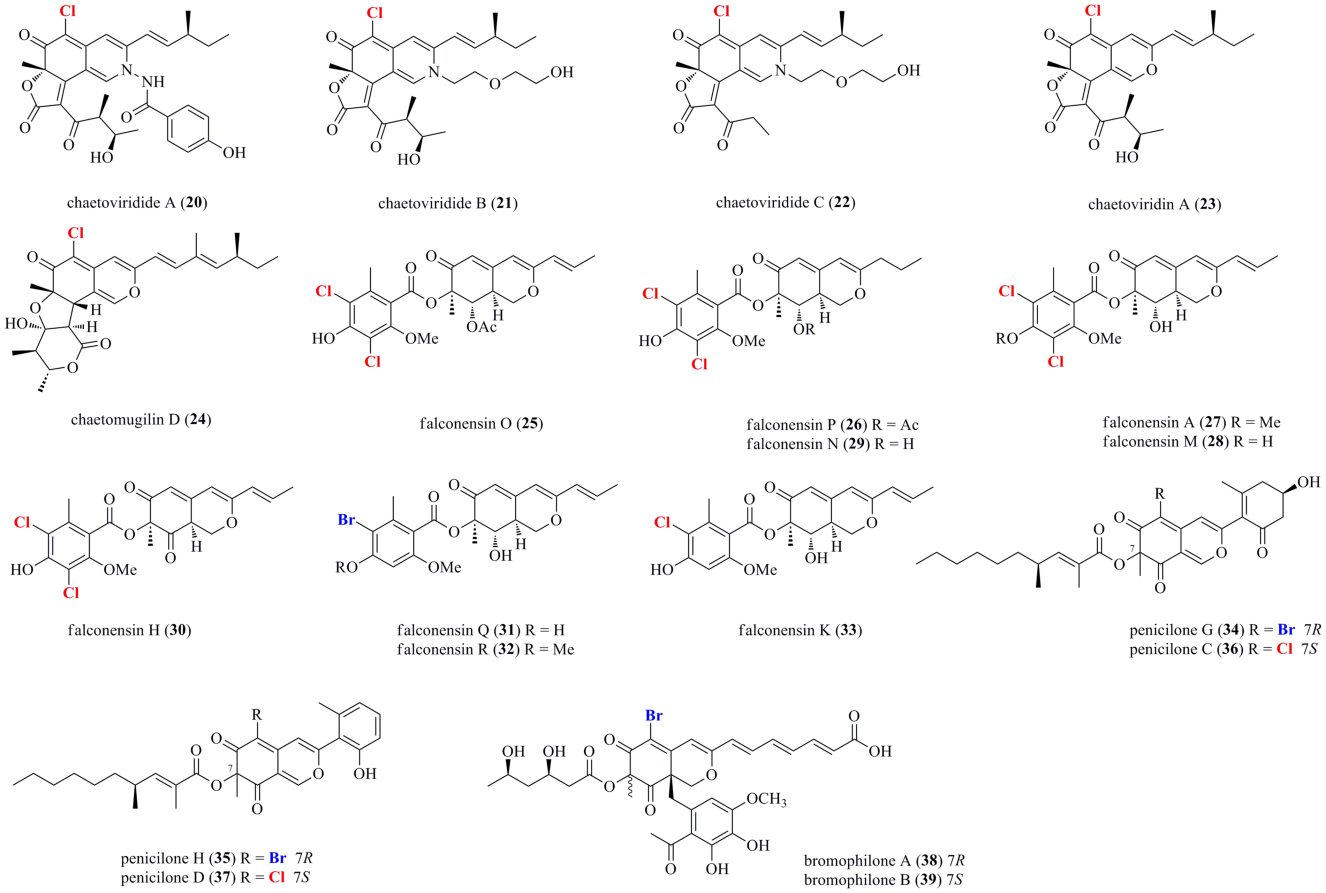
Halogenated azaphilones from marine-derived fungi (Continue) (**20–39**).

Thirty-nine halogenated azaphilones featured an oxabicyclic core were isolated from marine-derived fungi (Figures 1, 2; Table 1). Ten chlorinated azaphilones (**1**–**10**) including eight new nitrogenated azaphilones (**1**–**8**) were isolated from the deep-sea-derived fungus *Chaetomium globosum* MP4-S01-7 (Wang et al., 2020). Compounds **1**–**4** belong to *N*-(3,7-dimethyl-2,6-octadienyl) azaphilone polyketides, while compounds **5**–**8** are *N*-(3-methyl-2-butenyl) azaphilones. Most of them showed strong cytotoxic activity against the human gastric cancer MGC803 and AGS cell lines with IC_50_ values ranging from 0.12 to 10 μM. Importantly, compounds **1**, **2**, and **5**, in particularly, demonstrated the strongest activity at a nanomole level. In-depth mechanism study revealed that **2** arrested gastric cancer MGC803 and AGS cells in the G1 phase, while **1** and **2** induced apoptosis of both cells in a concentration-dependent manner. Eight chlorinated azaphilones, including five new ones **11**–**15** as well as three known analogs **16**–**18** were isolated from the deep-sea-derived fungus *Phomopsis tersa* FS441 (Chen et al., 2021). It should be pointed out that, compound **12**, which featured a cleaved tetrahydrofuranyl ring, possesses the novel 6/6–6 carbon framework. Moreover, compounds **14** and **15** are characterized as a pair of diastereomers with a characteristic epoxide ring, which are uncommon in azaphilones. In the cytotoxic assay, the new compounds **14** and **15** showed potent cytotoxicity against MCF-7, SF-268, and A549 cell lines with the IC_50_ values of 5.4–8.3 μM (compared with the positive control cisplatin, IC_50_ of 1.6–3.3 μM). Chemical investigations of *Chaetomium* sp. NA-S01-R1, which was isolated from the deep-sea seawater sample, yielded four new chlorinated azaphilone pigments (**19**–**22**) and two known ones (**23**–**24**; Wang et al., 2018). Compound **19** is a novel azaphilone bearing a fused tetrahydrofuran and *δ*-lactone moiety. The new azaphilones **20** and **21** exhibited antibacterial activities against aquatic pathogenic bacteria *Vibrio rotiferianus* and *V. vulnificus*, with MIC values of 7.3 and 7.4 μg/ml, respectively, while compounds **19**, **21** and **22** were found to possess anti-methicillin resistant *Staphylococcus aureus* activity with MIC values ranging from 7.3 to 7.8 μg/ml (chloramphenicol as the positive control with an MIC value of 7.6 μg/ml). Moreover, compound **20** showed cytotoxic activity against the HepG2 cell line with an IC_50_ value of 3.9 μM. The marine-derived fungus *Aspergillus falconensis*, when cultured on solid rice medium containing 3.5% NaCl, yielded two new chlorinated azaphilones **25** and **26** as well as four known derivatives **27**–**30** (El-Kashef et al., 2020). Then, replacing NaCl with 3.5% NaBr induced accumulation of two additional brominated azaphilones **31** and **32** and a known analog **33**. All of these compounds were examined for their nuclear factor kappa B (NF-κB) inhibitory activity in the triple negative breast cancer cell line MDA-MB-231. As a result, compounds **25** and **27**–**32** showed NF-κB inhibitory activity against the MDA-MB-231 cell line with IC_50_ values ranging from 11.9 to 72.0 μM. The mangrove rhizosphere soil-derived fungus *Penicillium janthinellum* HK1-6 was found to produce chlorinated azaphilones **36** and **37** (Chen et al., 2019). Cultivation of this fungal strain with NaBr instead of sea salt led to the isolation of two new brominated azaphilones **34** and **35**. Structurally, compounds **34**–**37** have a 7-*O*-2′,4′-dimethyldec-2′-enoyl side chain. The NaBr-induced brominated azaphilones **34** and **35** possess the opposite configuration at C-7 to the chlorinated analogs **36** and **37**. The brominated **35** exhibited antibacterial activity against the Gram-positive bacteria including both antibiotic-resistant (methicillin-resistant *Staphylococcus aureus* and vancomycin-resistant *Enterococcus faecium*) and antibiotic-susceptible (*S. aureus* and *E. faecalis*) strains with MIC values of 3.13–12.5 μg/ml. Fermentation of the fungus *P. canescens* 4.14.6a obtained from the Mediterranian sponge *Agelas oroides* with the addition of 5% NaBr yielded two new brominated azaphilones **38** and **39** (Frank et al., 2019). Compounds **38** and **39**, which represent the first azaphilones with a benzene moiety and the pyranoquinone skeleton *via* a methylene group, were exclusively produced when the fungus was cultivated with NaBr. Compound **39** exerted mild cytotoxicity against the mouse lymphoma cell line L5178Y (IC_50_ = 8.9 μM) and the human ovarian cancer cell line A2780 (IC_50_ = 2.7 μM), while its epimer **38** was relatively less active.

**Table 1:** Halometabolites isolated from marine-derived fungi (**1–145**).

Compounds	Fungus	Source	Biological activities	Reference
**1**–**10**	*Chaetomium globosum* MP4-S01-7	Deep-sea water sample (4,300 m)	Cytotoxic activity	Wang et al. (2020)
**11**–**18**	*Phomopsis tersa* FS441	Deep-sea sediment sample (3,000 m)	Cytotoxic activity	Chen et al. (2021)
**19**–**24**	*Chaetomium* sp. NA-S01-R1	Deep-sea seawater sample (4,050 m)	Antimicrobial and cytotoxic activities	Wang et al. (2018)
**25**–**33**	*Aspergillus falconensis*	Marine sediment	Anti-inflammatory activity	El-Kashef et al. (2020)
**34**–**37**	*Penicillium janthinellum* HK1-6	Mangrove rhizosphere soil	Antimicrobial activity	Chen et al. (2019)
**38**–**39**	*P. canescens* 4.14.6a	Sponge *Agelas oroides*	Cytotoxic activity	Frank et al. (2019)
**40**–**47**	*Pestalotiopsis colombiensis*	Sponge *Axinella* sp.	-	Lei et al. (2020)
**48**	*Pestalotiopsis* sp.	Soft coral *Sarcophyton* sp.	Antibacterial activity	Wei et al. (2013)
**49**	*Chaetomium* sp.	Marine algae	Antiprotozoal activity	Pontius et al. (2008)
**50**–**58**	*Aspergillus* sp. SCSIO F063	Marine sediment sample (1,451 m)	Cytotoxic activity	Huang et al. (2012)
**59**–**60**	*P. canescens* 4.14.6a	Sponge *Agelas oroides*	No cytotoxic activity	Frank et al. (2019)
**61**–**64**	*A. alliaceus*	Marine algae	Cytotoxic activity	Mandelare et al. (2018)
**65**–**66**	*Phoma* sp. 135	Sponge *Ectyplasia perox*	-	Elsebai and Ghabbour (2016)
**67**–**68**	*Spiromastix* sp. MCCC 3A00308	Marine sediment (2,869 m)	Antibacterial activity	Niu et al. (2021)
**69**	*A. niger*	Marine mudflat	Antioxidant activity	Leutou et al. (2016)
**70**	*A. ochraceus*	Marine red alga *Chondria crassicualis*	Antioxidant activity	Yun et al. (2013)
**71**–**72**	*Pseudallescheria boydii*	Marine starfish *Acanthaster planci*	-	Yan et al. (2015)
**73**	*P. canescens* 4.14.6a	Sponge *Agelas oroides*	No cytotoxic activity	Frank et al. (2019)
**74**	*Pleosporales* sp. HDN1811400	Marine sediment	Antibacterial activity	Han et al. (2021)
**75**	*Cladosporium cladosporioides* HDN14-342	Deep-sea sediment (3,471 m)	Cytotoxic activity	Zhang et al. (2016)
**76**	*C. cladosporioides* 8–1	Cold-seep	Antimicroalgal activity	Li et al. (2022)
**77**–**78**	*A. sydowii*	Marine alga *Acanthophora spicifera*	-	Teuscher et al. (2006)
**79**	*Roussoella* sp. DLM33	Source ungiven	-	Ferreira et al. (2015)
**80**–**81**	*P. terrestre*	Marine sediments	No cytotoxic activity	Li et al. (2011)
**82**	*Trichoderma harzianum* (XS-20090075)	Soft coral	No antifouling activity	Yu et al. (2021)
**83**–**85**	*Phoma* sp.135	Sponge *Ectyplasia perox*	Antibacterial activity	Elsebai et al. (2018)
**86**–**87**	*P. terrestre*	Marine sediments	Cytotoxic activity	Li et al. (2011)
**88**–**89**	*Cochliobolus lunatus* (TA26-46)	Sea anemone *Palythoa haddoni*	No cytotoxic activity	Zhang W. et al. (2014)
**90**–**91**	Unidentified	Marine alga *Gracillaria verrucosa*	-	Li et al. (2004)
**92**	*Tryblidiopycnis* sp. 4,275	Mangrove Kandelia	-	Huang et al. (2006)
**93**	*Penicillium* sp. MMS351	Seawater sample	Cytotoxic activity	Vansteelandt et al. (2013)
**94**–**97**	*Penicillium* sp. PR19N-1	Marine sludge	Cytotoxic activity	Wu et al. (2013)
**98**	*T. harzianum* (XS-20090075)	Soft coral	No antimicrobial activity	Shi et al. (2020)
**99**–**100**	*Penicillium* sp. SCS-KFD09	Marine worm *Sipunculus nudus*	Antiviral activity	Kong et al. (2017)
**101**	*A. nidulans* EN-330	Marine alga *Polysiphonia scopulorum*	Antimicrobial activity	Zhang et al. (2015)
**102**–**107**	*Malbranchea aurantiaca*	Marine invertebrate	-	Watts et al. (2011)
**108**–**110**	*Phomopsis* sp. QYM-13	Mangrove *Kandelia candel*	Cytotoxic activity	Chen et al. (2022)
**111**–**115**	*Trichoderma* sp. TPU199	Marine alga	-	Yamazaki et al. (2020)
**116**	*A. alliaceus*	Marine alga	-	Mandelare et al. (2018)
**117**–**119**	*A. flavipes* 164,013	Sponge	Enzyme inhibitory activity	Jiao et al. (2020)
**120**	*T. harzianum* (XS-20090075)	Soft coral	No antifouling activity	Yu et al. (2021)
**121**	*Graphostroma* sp. MCCC 3A00421	Deep-sea hydrothermal sulfide	No antifood allergic activity	Niu et al. (2018)
**122**–**123**	*P. canescens* 4.14.6a	Sponge *Agelas oroides*	No cytotoxic activity	Frank et al. (2019)
**124**–**130**	*A. unguis* GXIMD 02505	Coral *Pocillopora damicornis*	Anti-osteoclastogenic and antibacterial activity	Zhang et al. (2022)
**131**–**133**	*Spiromastix* sp. MCCC 3A00308	Marine sediment (2,869 m)	Antibacterial activity	Niu et al. (2021)
**134**–**135**	*A. unguis*	Seaweed	Antimicrobial and larvicidal activity	Zhang Y. et al. (2014)
**136**	*P. citreonigrum* XT20-134	Deep-sea sediment (2,910 m)	Cytotoxic activity	Tang et al. (2019)
**137**–**143**	*Acremonium sclerotigenum* GXIMD 02501	Coral *Pocillopora damicornis*	Anti-osteoclastogenic activity	Lu et al. (2022)
**144**–**145**	*Aspergillus* sp.	Marine alga *Ishige okamurae*	Antioxidant activity	Leutou et al. (2013)

#### Benzophenones

**Figure 3:**
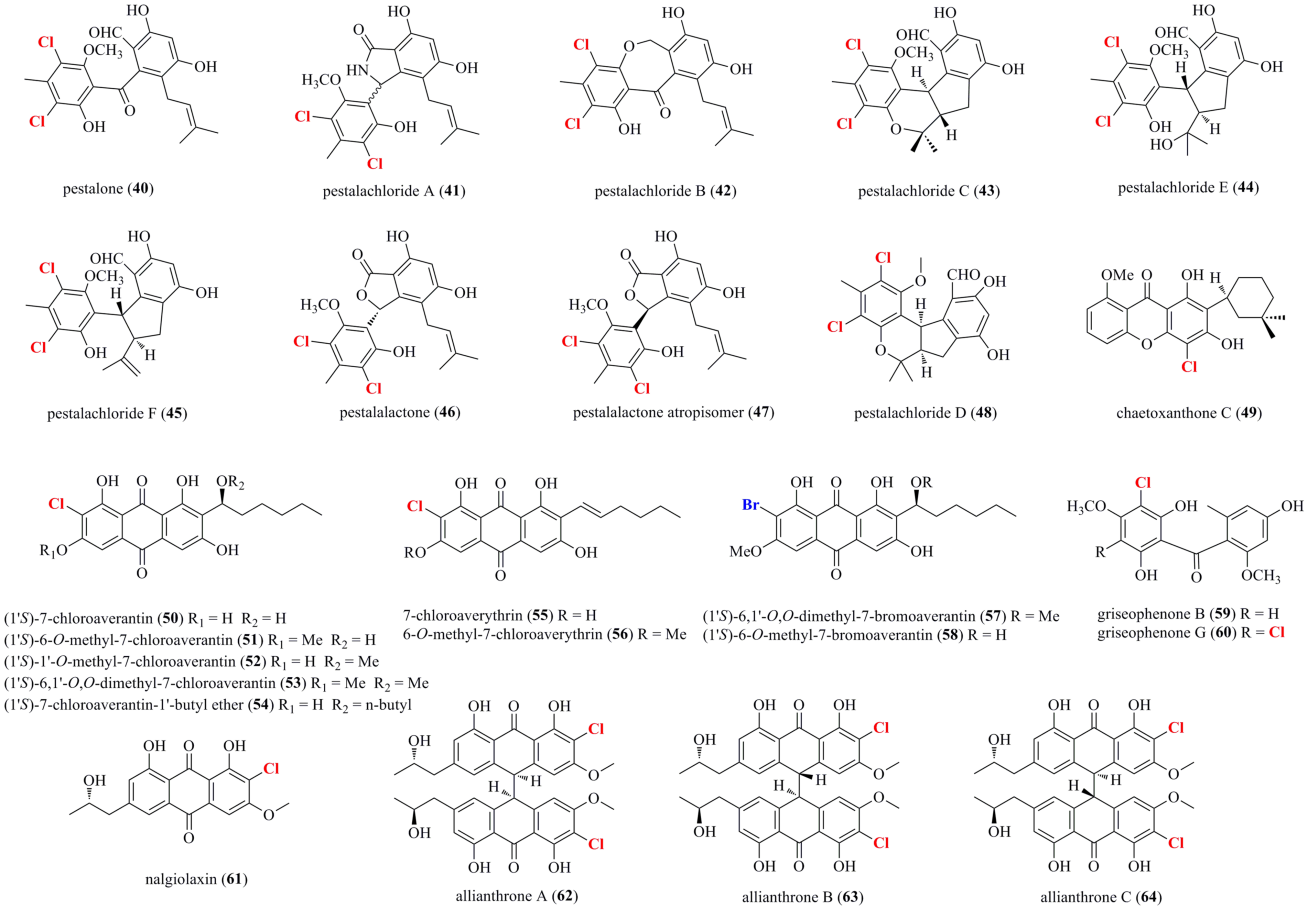
Halogenated benzophenones from marine-derived fungi (**40–64**).

As shown in Figure 3, 25 halogenated benzophenones (**40**–**64**) were isolated from marine-derived fungi. A chemical survey of the sponge-associated fungus *Pestalotiopsis colombiensis* yielded eight chlorinated benzophenone derivatives **40**–**47**, which were isolated from this fungal species for the first time (Lei et al., 2020). These compounds, exclusively isolated from the genus *Pestalotiopsis* and never found in other genus, possess a great significance in the chemotaxonomic study of *Pestalotiopsis*. Therefore, they could be regarded as important chemotaxonomic markers for the genus of *Pestalotiopsis*. A new chlorinated benzophenone derivative **48** was isolated from the soft coral-derived fungus *Pestalotiopsis* sp. (Wei et al., 2013). Compound **48** demonstrated antibacterial activities against *Escherichia coli*, *V. anguillarum*, and *V. parahaemolyticus* with MIC values of 5.0, 10.0 and 20.0 μM, respectively. A new chlorinated xanthone **49** substituted with a tetrahydropyran ring was isolated from the marine-derived fungus *Chaetomium* sp. (Pontius et al., 2008). Compound **49** showed moderate antiprotozoal activity against *Trypanosoma cruzi* with an IC_50_ value of 1.5 μg/ml. Metabolomic investigations on the marine-derived fungus *Aspergillus* sp. SCSIO F063 unveiled seven new chlorinated anthraquinones **50**–**56** (Huang et al., 2012). Futhermore, when the fungus was fermented with 3% NaBr, two new brominated anthraquinones **57** and **58** were additionally isolated. Interestingly, no iodinated secondary metabolites were observed when the fungus was fermented with NaI. Among these metabolites, only compound **51** moderately inhibited the growth of three human tumor cell lines, SF-268, MCF-7, and NCI-H460, with IC_50_ values of 7.11, 6.64, and 7.42 μM, respectively. The above-mentioned fungal strain *P. canescens* 4.14.6a cultured in sea salt produced compounds **59** and **60** (Frank et al., 2019). Metabolic studies on two different developmental stages, the vegetative stage (asexual morph) and the sexual stage (sclerotial morph), of the marine algal-derived fungus *A. alliaceus* were performed (Mandelare et al., 2018). As a result, the asexual morph of *A. alliaceus* produced a chlorinated anthraquinone **61**, whereas three chlorinated bianthrones **62**–**64** were generated by the coculture of the asexual and sclerotial morph of *A. alliaceus*. Compound **62** was active against the HCT-116 colon carcinoma and SK-Mel-5 skin cancer cell lines with IC_50_ values of 9.0 and 11.0 μM, respectively.

#### Coumarin-/chromone/pyran-/furan-derived polyketides

**Figure 4:**
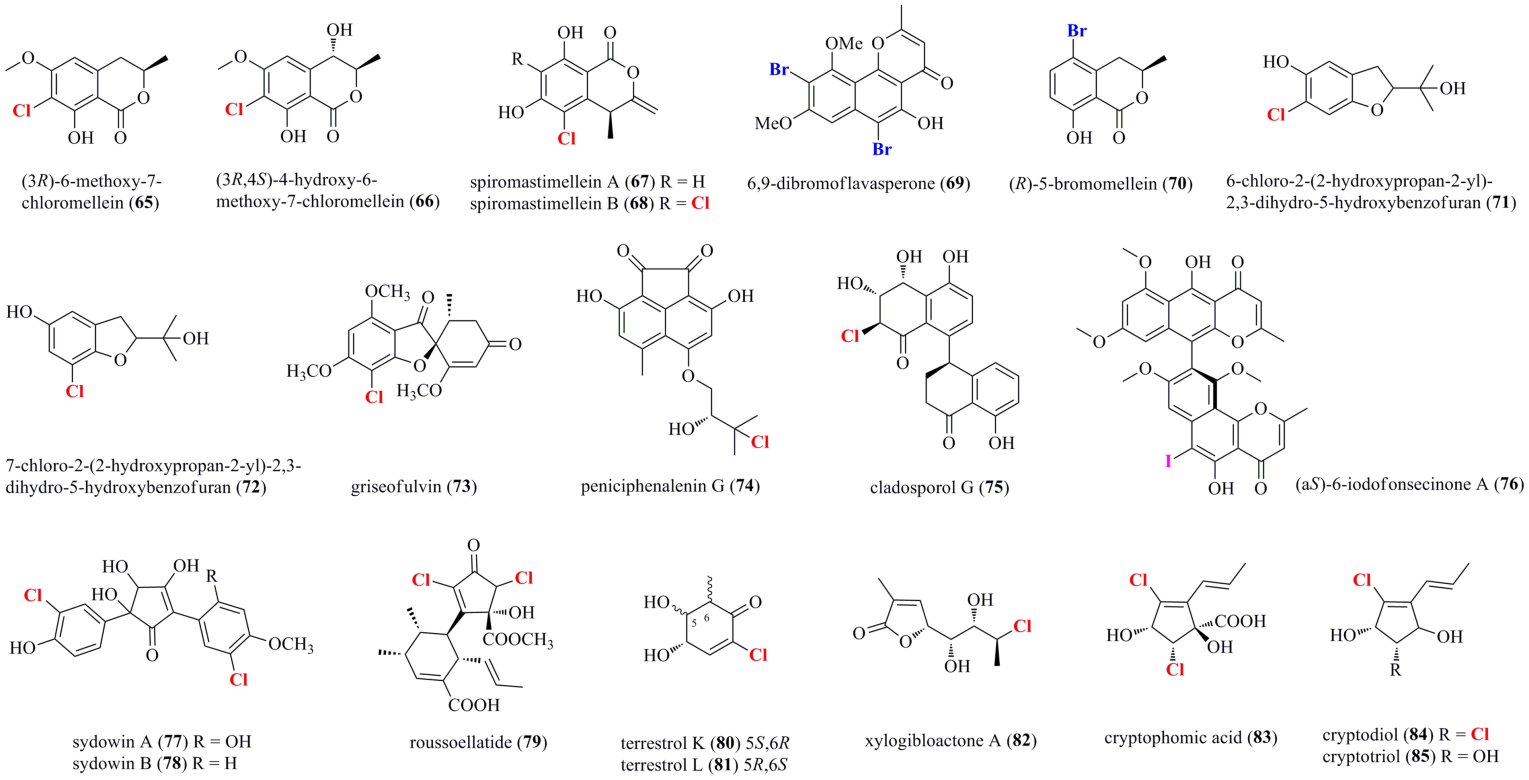
Halogenated coumarin−/chromone/pyran−/furan-derived polyketides from marine-derived fungi (**65–85**).

Diverse coumarin-/chromone/pyran-/furan-derived polyketides (**65**–**85**) isolated from marine-derived fungi are shown in Figure 4. Two chlorinated dihydro-isocoumarin derivatives **65** and **66** were isolated from the marine-derived fungus *Phoma* sp. 135 (Elsebai and Ghabbour, 2016). Two new chlorinated isocoumarins **67** and **68** with an exomethylene group at C-3 were isolated from a deep-sea-derived fungus *Spiromastix* sp. MCCC 3A00308 (Niu et al., 2021). The dichlorinated isocoumarin **68** showed higher antibacterial activity (*Bacillus thuringiensis* and *B. subtilis*, with an MIC value of 4 μg/ml) than the monochlorinated **67**. The addition of metal bromides, NaBr and CaBr_2_, to the medium of marine-mudflat-derived fungus *A. niger* induced the production of a new brominated naphthopyranone **69** (Leutou et al., 2016), while the addition of NaBr to a marine-derived *A. ochraceus* led to the induced production of a new brominated isocoumarin **70** (Yun et al., 2013). Compounds **69** and **70** displayed strong radical scavenging activity against DPPH with IC_50_ values of 21 and 24 μM, respectively. Two new chlorinated benzofuran derivatives, **71** and **72**, were isolated from the marine starfish-derived fungus *Pseudallescheria boydii* (Yan et al., 2015). A chlorinated griseofulvin-type spirocyclic polyketide **73** was isolated from *P. canescens* 4.14.6a (Frank et al., 2019). A new phenalenone **74**, representing the first example of chlorinated acenaphthenquinone derivative, was characterized from the marine sediment-derived fungus *Pleosporales* sp. HDN1811400 (Han et al., 2021). Compound **74** displayed higher inhibitory activity against MRCNS (MIC = 25.0 μM) and MRSA (MIC = 12.5 μM) than the positive control ciprofloxacin (MICs of 25.0 and > 50 μM, respectively), suggesting the high potential of these heptaketide phenalenones as lead compounds for drug-resistant pathogens. A new naturally occurring 8–4′ linkage 1-tetralone dimeric derivative **75** was isolated from the deep-sea derived fungus *Cladosporium cladosporioides* HDN14-342 (Zhang et al., 2016). Compound **75**, which represents the first halogenated cladosporol derivatives, showed cytotoxicity against HeLa, K562, and HCT-116 cell lines with IC_50_ values of 3.9, 8.8, and 19.4 μM. An unexpected iodinated dimeric naphtho-γ-pyrone **76** was obtained from the marine cold-seep fungus *C. cladosporioides* 8–1 (Li et al., 2022). Compared to chlorine- and bromine-containing compounds, iodine-bearing metabolites are rarely encountered. Compound **76** displayed potent antimicroalgal activity against the marine microalgae *Prorocentrum minimum* with an IC_50_ value being 0.61 μg/ml, compared with the positive control CuSO_4_ (IC_50_ = 2.4 μg/ml). Two new chlorinated cyclopentanoids **77** and **78** were isolated from *A. sydowii*, an endophyte associated with the marine alga *Acanthophora spicifera* (Teuscher et al., 2006). Both compounds are structurally related hydroxylated 2,5-diarylcyclopentenones, which have hitherto only been isolated from higher basidiomycetes. A novel dichlorinated compound **79** having an unprecedented polyketide skeleton was isolated from the marine-derived fungus *Roussoella* sp. DLM33 (Ferreira et al., 2015). Stable isotope feeding experiments revealed a complicated biosynthetic origin of **79** by Favorskii rearrangements in individual pentaketides before being linked *via* an intermolecular Diels−Alder reaction. Two new chlorinated quasi-precursors of sorbicillinoid-type polyketides, **80** and **81**, were isolated from the marine sediment-derived fungus *P. terrestre* (Li et al., 2011). A furan lactone **82** was isolated from the soft coral-derived fungus *Trichoderma harzianum* (XS-20090075) cultured with rice medium (Yu et al., 2021). Chromatographic separation of the marine-derived fungus *Phoma* sp.135 resulted in the characterization of three new chlorinated cyclopentene derivatives **83**–**85** (Elsebai et al., 2018). Compounds **83**–**85** showed weak antimicrobial activity against *E. coli*, *Bacillus subtilis*, *Mycobacterium phlei*, and *S. aureus*, with MIC values ranging from 10 to 35 μM.

#### Other polyketides

**Figure 5:**
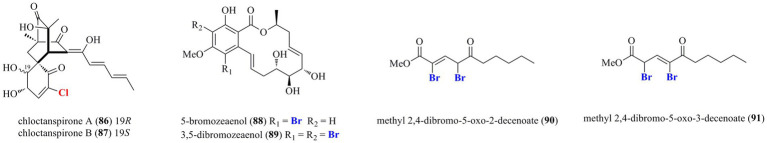
Other halogenated polyketides from marine-derived fungi (**86–91**).

As shown in Figure 5, compounds **86** and **87**, two novel chlorinated sorbicillinoids possessing an unprecedented bicyclo[2.2.2]octane-2-spiro cyclohexane skeleton, were isolated from *P. terrestre* (Li et al., 2011). Compounds **86** and **87** are identified as the first occurrence of spiro cyclohexane-containing and chlorinated sorbicillinoids. Interestingly, **86** was more active against HL-60 cell line with an IC_50_ value of 9.2 μM than **87** (IC_50_ = 37.8 μM), indicating that the stereochemistry may influence the cytotoxic activity. Chemical epigenetic modification, a promising approach to manipulate the silent fungal genes, was used to the marine-derived fungus *Cochliobolus lunatus* (TA26-46) with histone deacetylase inhibitors, led to the isolation and identification of two new brominated 14-membered resorcylic acid lactones **88** and **89** (Zhang W. et al., 2014). It should be noted that both compounds, which were identified as the first examples of brominated resorcylic acid lactones, were exclusively isolated *via* epigenetic modifying agents. Finally, two new dibrominated alkenoates **90** and **91** were isolated from an unidentified fungus (Li et al., 2004).

### Halogenated terpenoids from marine-derived fungi

**Figure 6:**
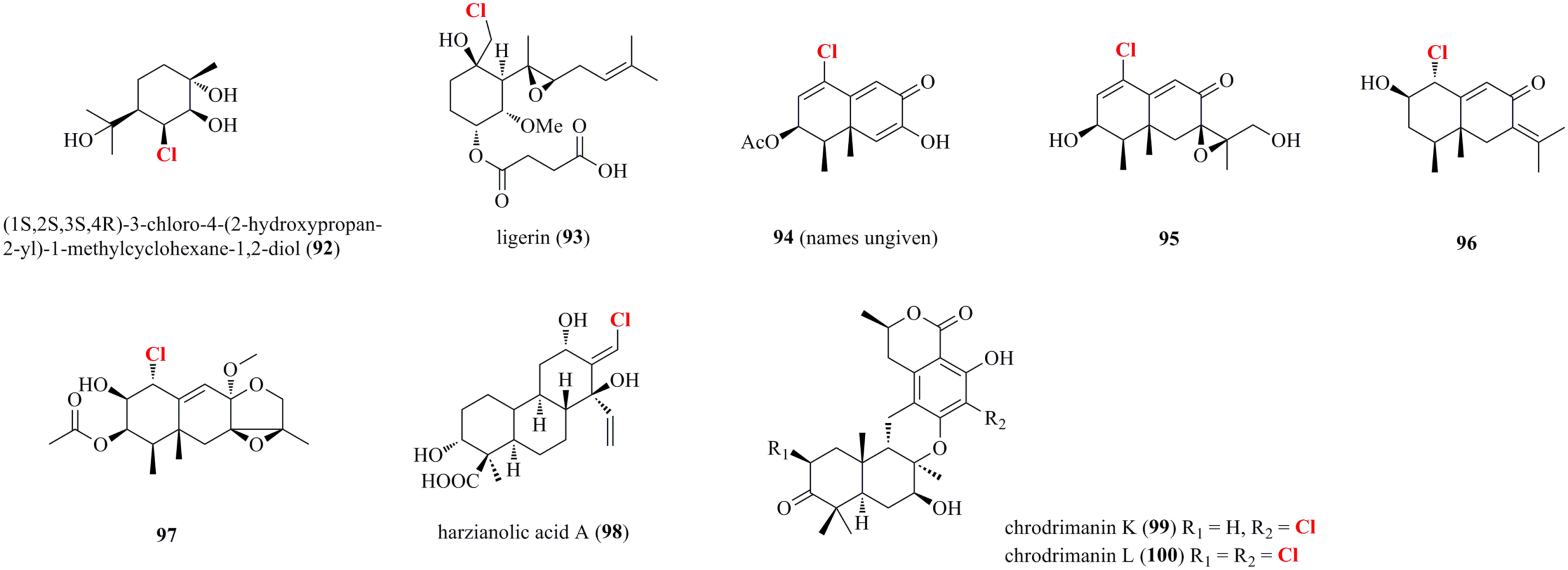
Halogenated terpenoids from marine-derived fungi (**92–100**).

Diverse halogenated terpenoids isolated from marine-derived fungi, including one monoterpene **92**, five sesquiterpenoids **93**–**97**, one diterpenoid **98**, and two meroterpenoids **99**–**100**, are shown in Figure 6. A new chloro-monoterpene **92** was isolated from the mangrove-sourced endophytic fungal strain *Tryblidiopycnis* sp. 4,275 (Huang et al., 2006). A new chlorinated sesquiterpenoid **93** was obtained from the marine-derived *Penicillium* strain MMS351 (Vansteelandt et al., 2013). **93** is elucidated as an analog of fumagillin, a sesquiterpene esterified by a deca-2,4,6,8-tetraenedioic acid and functionalized by a spiro-epoxide fused with the cyclohexane ring. Compound **93** showed potent antiproliferative activity against the osteosarcoma cell line POS1 with an IC_50_ value of 117 nM. Four new chlorinated eremophilane-type sesquiterpenes **94**–**97** were obtained from the deep-sea derived fungus *Penicillium* sp. PR19N-1 (Wu et al., 2013). Compound **94**, which is identified as a trinor-eremophilene core with an 8-oxo-1(2),9(10)-diene unit, was found to possess modest cytotoxic activity against HL-60 and A549 cell lines with IC_50_ values of 11.8 and 12.2 μM, respectively. A new chlorinated cleistanthane-type diterpenoid **98** was isolated from the soft coral-derived fungus *T. harzianum* (XS-20090075) cultured with 10 μM sodium butyrate (Shi et al., 2020). The cleistanthane-type diterpenoid, arisen owing to chemical epigenetic modification, was discovered from genus *Trichoderma* for the first time. Isolation of the marine worm (*Sipunculus nudus*)-derived fungus *Penicillium* sp. SCS-KFD09 afforded two new previously unreported chlorinated meroterpenoids **99** and **100** (Kong et al., 2017). Both meroterpenoids possess a drimane-type sesquiterpenoid substructure fused with an isochromanone moiety. Compound **99** showed strong antiviral activity against influenza A virus (H1N1) with an IC_50_ value of 74 μM (ribavirin as positive control with an IC_50_ of 103 μM).

### Halogenated alkaloids from marine-derived fungi

**Figure 7:**
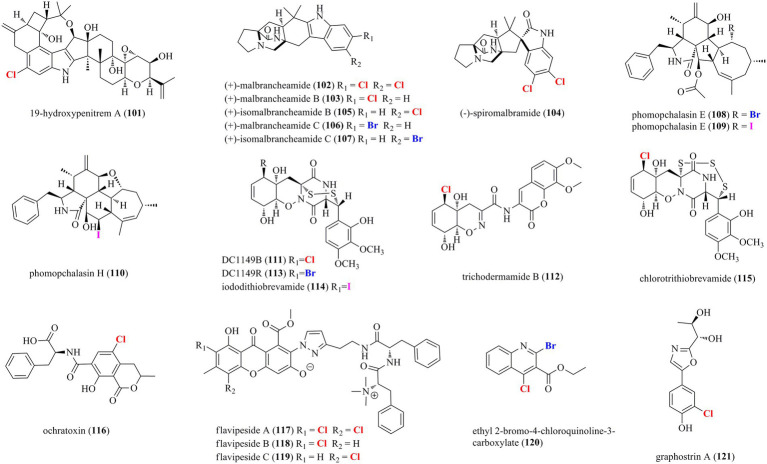
Halogenated alkaloids from marine-derived fungi (**101–121**).

A total of 21 halogenated alkaloids (**101**–**121**, Figure 7) were isolated from marine-derived fungi. A new chlorinated indole-diterpenoid **101** was isolated from the algal-endophytic fungus *A. nidulans* EN-330 (Zhang et al., 2015). Compound **101** inhibited the growth of brine shrimp (*Artemia salina*) with an LD_50_ value of 3.2 μM. Moreover, it also displayed antimicrobial activities against human- (*E. coli* and *S. aureus*) and aqua- (*Edwardsiella tarda* and *V. anguillarum*) pathogens with MIC values of 16–64 μg/ml. The chlorine-substitution may enhance bioactivities to some degree. Prenylated indole alkaloids possessing a characteristic bicyclo[2.2.2]diazaoctane or diketopiperazine ring are a diverse group of fungal secondary metabolites for biosynthetic investigations (Zhang et al., 2019). A systematic isolation of *Malbranchea aurantiaca*, obtained from an unidentified marine invertebrate, provided six new halogenated prenylated indole alkaloids **102**–**107** (Watts et al., 2011). Structurally, all of the isolated compounds are identified as prenylated indole alkaloids containing a halogenated indole ring and the bicyclo[2.2.2]diazaoctane skeleton. Compounds **102**–**105** were isolated in normal artificial seawater medium, while two brominated **106** and **107** were produced by modifying the solid growth medium with NaBr. Inspired by OSMAC approach, the mangrove-derived fungus *Phomopsis* sp. QYM-13 was cultured with the addition of NaBr or KI to afford halogen-substituted metabolites. As a result, a new brominated cytochalasin **108** and two new iodinated cytochalasins **109** and **110** were isolated from this strain treated with 3% NaBr and 3% KI, respectively (Chen et al., 2022). Compounds **109** and **110** represent the first iodinated cytochalasins. The brominated **108** displayed selective cytotoxicity to MDA-MB-435 cell line with an IC_50_ value of 7.4 μM. Research into the fungus *Trichoderma* sp. TPU199 derived from a red alga yielded a series of new epipolythiodiketopiperazines **111**–**115** with a sulfide bridge (–S–, –SS–, –SSS–, or –SSSS–) between the α- and β-positions of two amino acid residues (Yamazaki et al., 2020). This fungal strain afforded the halogenated **111**, **113**, and **114**, when fermented with 3% NaCl, NaBr, and NaI, respectively. Moreover, compound **115**, the first trisulfide derivative, was induced by cultivation of this strain with DMSO. A chlorinated mycotoxin **116** was isolated from sclerotial morph of *A. alliaceus* (Mandelare et al., 2018). Three unprecedented chlorinated PKS-NRPS hybrid metabolites **117**–**119** were isolated from the marine sponge symbiotic fungus *A. flavipes* 164,013 (Jiao et al., 2020). These compounds consisting of a chlorinated xanthone, an aminoethyl-modified pyrazol, and a methylated dipeptide represent a new structural family of PKS-NRPS hybrid metabolites. Compounds **117**–**119** showed significant inhibitory activity on pancreatic lipase with IC_50_ values of 0.23, 0.07, and 0.14 μM, respectively, which were 6–21 times more potent than that of the positive control kaempferol (IC_50_ = 1.50 μM). A new brominated chloroquinoline **120** was isolated from the fungus *T. harzianum* (Yu et al., 2021). **120** was isolated as the first halogenated quinoline derivative from the genus *Trichoderma*. A novel chlorinated alkaloid **121** featuring a rare oxazole moiety was isolated from the hydrothermal fungus *Graphostroma* sp. MCCC 3A00421 (Niu et al., 2018).

### Halogenated phenolic derivatives from marine-derived fungi

**Figure 8:**
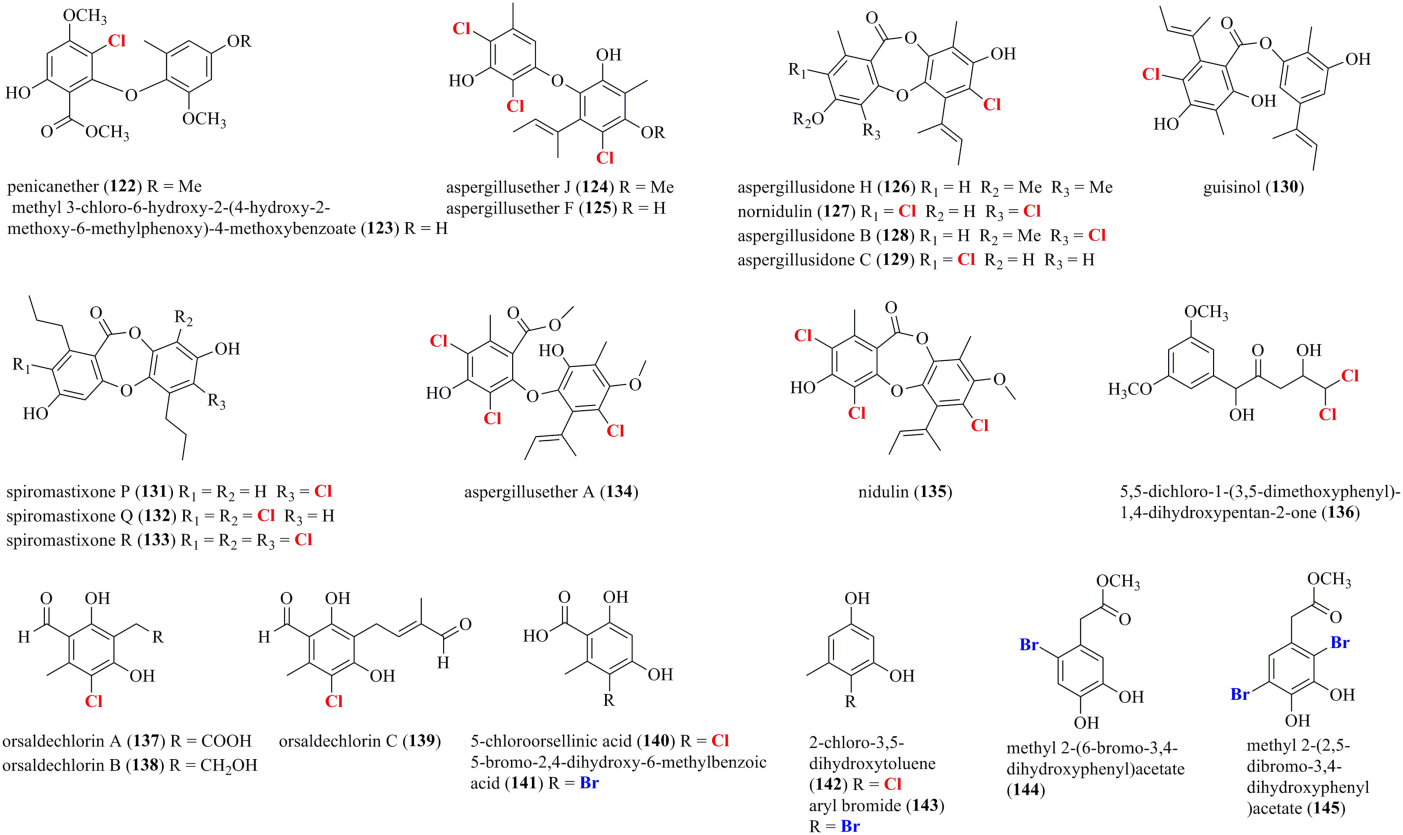
Halogenated phenolic derivatives from marine-derived fungi (**122–145**).

Figure 8 presents a total of 24 halogenated phenolic derivatives (**122**–**145**) isolated from marine-derived fungi. Two chlorinated diphenyl ethers, **122** and **123**, were isolated from the sponge-associated fungus *P. canescens* 4.14.6a (Frank et al., 2019). Seven chlorinated phenolic derivatives, including two diphenyl ethers (**124** and **125**), four depsidones (**126**–**129**), and one depside (**130**), were isolated from the coral-derived fungus *A. unguis* GXIMD 02505 (Zhang et al., 2022). Compounds **124**–**128** and **130** were found to inhibit lipopolysaccharide (LPS)-induced NF-κB in RAW 264.7 macrophages at a concentration of 20 μM. Most importantly, compounds **125** and **130**, acted as the most potent inhibitors, dose-dependently suppressed RANKL-induced osteoclast differentiation. In addition, compounds **124**, **125**, **127**, **129**, and **130** displayed moderate antibacterial activities against methicillin-resistant *S. aureus*, *Microbulbifer variabilis*, *Marinobacterium jannaschii*, and *V. pelagius* with the MIC values ranging from 2 to 64 μg/ml. Three new chlorinated depsidone-type compounds (**131**–**133**) were isolated from the deep-sea-derived *Spiromastix* fungus (Niu et al., 2021). Compound **133** was characterized as a tri-chlorinated derivative and possessed remarkable antibacterial activities against *S. aureus*, *Bacillus thuringiensis*, and *B. subtilis*, with MIC values of 0.5–1.0 μg/ml. Two tri-chlorinated depsidones **134** and **135** were isolated from a seaweed-derived *A. unguis* strain (Zhang Y. et al., 2014). Compound **135** strongly inhibited methicillin-resistant *S. aureus* (MIC = 4 μg/ml) and brine shrimp *Artemia larva* (LC_50_ = 2.8 μg/ml). A new dichlorinated compound **136** was isolated from the deep-sea sediment-derived fungus *P. citreonigrum* XT20-134 (Tang et al., 2019). Compound **136** possessed promising cytotoxicities against the human hepatoma tumor cell Bel7402 and the human fibrosarcoma tumor cell HT1080, with IC_50_ values of 13.14 and 16.53 μM, respectively. Seven halogenated phenolic derivatives, including three new chlorinated orsellinic aldehyde derivatives **137**–**139**, two orsellinic acids (chlorinated **140** and brominated **141**), and two phenols (chlorinated **142** and brominated **143**), were isolated from the coral-associated fungus *Acremonium sclerotigenum* GXIMD 02501 (Lu et al., 2022). Compounds **137**, **138**, **140**, and **143** showed certain inhibition of LPS-induced NF-κB activation in RAW 264.7 cells at 20 μM. Two new potent inhibitors (**137** and **138**) strongly suppressed RANKL-induced osteoclast differentiation. Finally, the addition of NaBr and CaBr_2_ in the fermentation of the marine-derived fungus *Aspergillus* sp. induced the production of two new brominated dihydroxyphenylacetic acid derivatives **144** and **145** (Leutou et al., 2013). Both compounds exerted strong DPPH scavenging activity with IC_50_ values of 14.2 and 12.1 μM.

## Induced production of halometabolites with different cultural conditions

**Figure 9:**
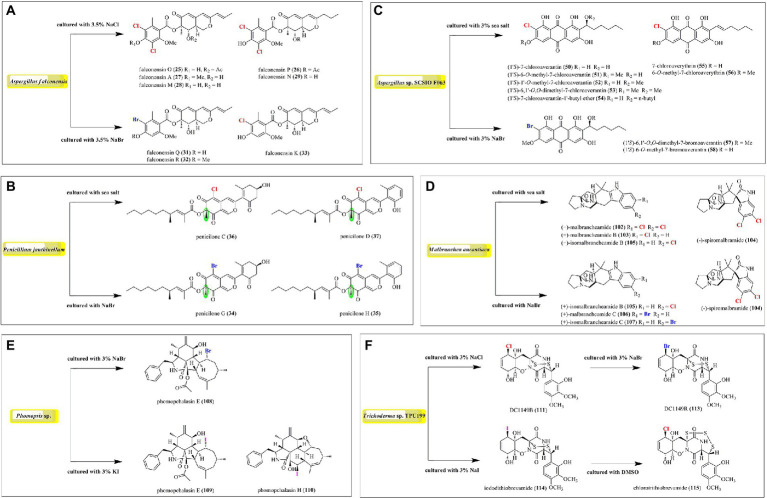
Induced production of halometabolites with different cultural conditions. **(A)** Azaphilones produced by Aspergillus falconensis; **(B)** Azaphilones produced by Penicillium janthinellum; **(C)** Anthraquinones produced by Aspergillus sp. SCSIO F063; **(D)** Prenylated indole alkaloids produced by Malbranchea aurantiaca; **(E)** Cytochalasin produced by Phomopsis sp. QYM-13; **(F)** Epipolythiodiketopiperazines produced by Trichoderma sp. TPU199.

In order to expand the structural diversity of the halometabolites from the marine-derived fungi, OSMAC (One Strain MAny Compounds) strategy was used to remodel the fungal metabolome and activate the cryptic biosynthetic pathways. Of all the isolated halometabolites from the marine-derived fungi, most of them are chlorinated (81.4%), then followed by brominated (15.9%), while iodinated compounds are rather rare (2.7%). It should be pointed out that the occurrence of halogenated metabolites depends on halogen salts in the fermentation of the producing fungi. It seems that most of the brominated and iodinated compounds are generated by the substitution of bromide and iodide ions for the chloride ion in cultivation (Figure 9). For example, fermentation of *A. falconensis* with 3.5% NaCl afforded chlorinated azaphilones **25**–**30**, while replacing NaCl with 3.5% NaBr induced the production of additional brominated azaphilones **31** and **32** (El-Kashef et al., 2020). Cultivation of *P. janthinellum* HK1-6 with sea salt and NaBr yielded chlorinated azaphilones **36**–**37** and brominated **34**–**35**, respectively (Chen et al., 2019). Interestingly, the NaBr-induced brominated **34**–**35** possess the opposite configuration at C-7 compared to the chlorinated analogs **36**–**37** cultured with normal sea salt condition. In addition to the chlorinated anthraquinones **50**–**56**, two brominated anthraquinones **57** and **58** were obtained from *Aspergillus* sp. SCSIO F063 by the substitution of 3% NaBr for sea salt (Huang et al., 2012). The authors also fermented the fungus with NaI; however, no iodinated metabolites were observed. The fungus *M. aurantiaca* produced chlorinated prenylated indole alkaloids **102**–**105**, when fermented in normal artificial seawater medium, while the brominated **106** and **107** were isolated from its culture broth in NaBr-containing medium (Watts et al., 2011). The fungus *Phomopsis* sp. QYM-13 cultured with the addition of 3% NaBr or 3% KI was found to produce a brominated cytochalasin **108** and two new iodinated cytochalasins **109** and **110**, respectively (Chen et al., 2022). Finally, the fungus *Trichoderma* sp. TPU199 afforded the halogenated **111**, **113**, **114**, and **115** when induced by cultivation of this fungal strain with 3% NaCl, 3% NaBr, 3% NaI, and DMSO, respectively (Yamazaki et al., 2020). These results indicated that the substitution of bromide or iodide ions for sea salt in the fermentation of the producing fungi may be an effective way to afford more intriguing halometabolites, expecially brominated and iodinated compounds, from the marine-derived fungi.

## Conclusions and future perspectives

**Figure 10:**
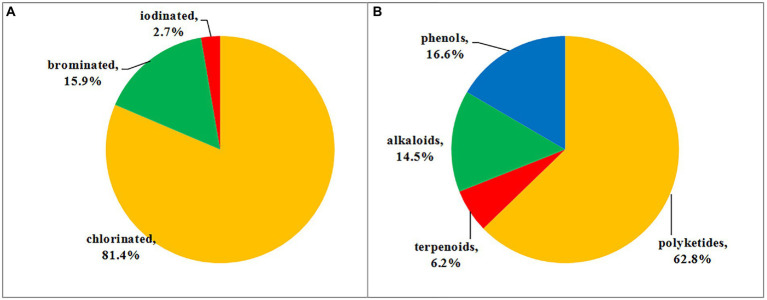
**(A)** Proportion of halometabolites from marine-derived fungi; **(B)** Structural classes of halometabolites.

Halometabolites are mainly produced by marine organisms due to the presence of chloride, bromine, and iodine ions in seawater. As previously discussed, among all of the halometabolites described herein, chlorination is the predominant modification, and then followed by bromination, while iodination is extremely rare. In this review, a total of 118 chlorinated (accounting for 81.4%), 23 brominated (15.9%), and four iodinated (2.7%) metabolites isolated from marine-derived fungi were summarized (Figure 10A). Marine fungi may possess the capability to oxidize chlorine more easily than bromide and iodine in the biosynthesis of these metabolites, thus the number of chlorinated compounds is quite higher than brominated and iodinated compounds. Moreover, these halometabolites possess a high structural diversity. The reported 145 halometabolites, shown in this review, are categorized into polyketides (**1**–**91**; including azaphilones **1**–**39**, benzophenones **40**–**64**, coumarin−/chromone/pyran−/furan-derived polyketides **65**–**85**, and other types of polyketides **86**–**91**), terpenoids (**92**–**100**), alkaloids (**101**–**121**), and phenolic derivatives (**122**–**145**). Structural classification of compounds based on biogenetic categories is unprecise, as many compounds are derived from mixed biosynthetic pathways. For example, compounds **1**–**6** are clearly classified as nitrogen-containing compounds. However, we categorize them as polyketides based on the biosynthetic origin of azaphilones. It is estimated that 62.8% of the reported halometabolites are polyketides (Figure 10B), especially azaphilones, which accounted for 42.9% of the reported halogenated polyketides. As for the halogenated alkaloids, a series of halogenated prenylated indole alkaloids **102**–**107** and epipolythiodiketopiperazines **111**–**115** were isolated and induced by the addition of additional halogen salts. Changing the cultural conditions will help to increase the chemical diversity of halometabolites produced by marine-derived fungi.

**Figure 11:**
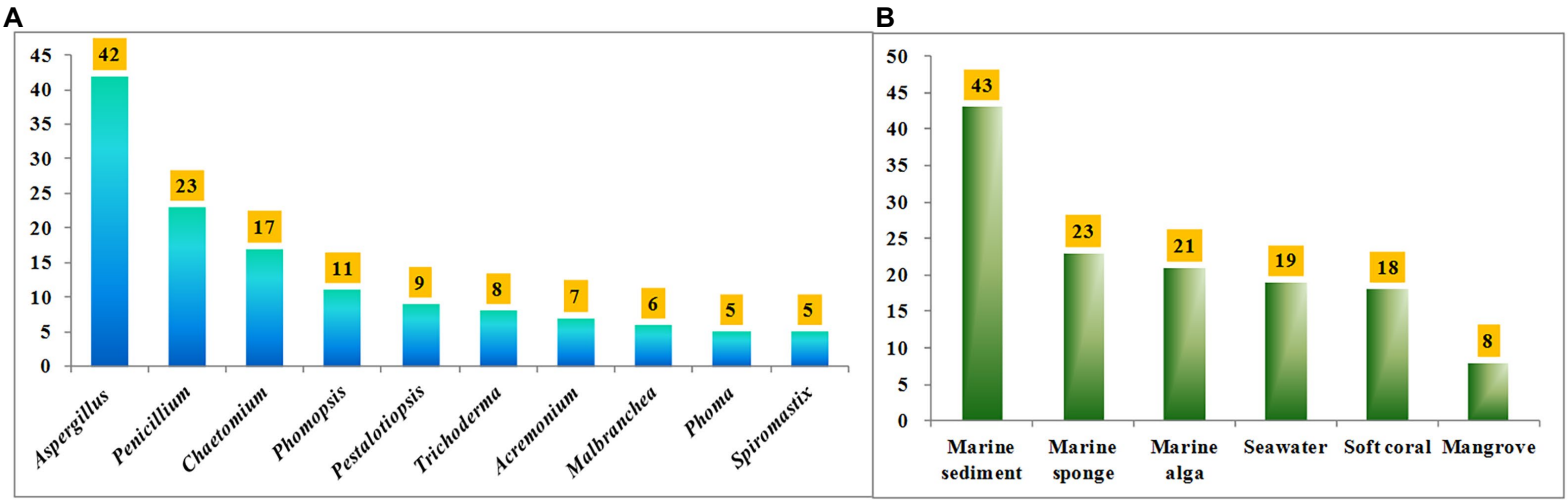
**(A)** Numbers of halometabolites from different marine-derived fungi; **(B)** Numbers of halometabolites from different sources of marine origins.

Halometabolites isolated from marine microorganisms are relatively unexplored compared with those from marine macroorganisms, such as algae, sponges, and soft corals. Marine-derived fungi have proven to be a precious house of bioactive secondary metabolites with novel structures. Table 1 shows a total of 17 genera of marine-derived fungi as producers of these halometabolites. Among them, the species belonging to genera *Aspergillus*, *Penicillium*, *Chaetomium*, *Phomopsis*, *Pestalotiopsis*, *Trichoderma*, *Acremonium*, *Malbranchea*, *Phoma*, and *Spiromastix* are the Top 10 producers, with 42, 23, 17, 11, 9, 8, 7, 6, 5, and 5 halometabolites being isolated, respectively (Figure 11A). In addition, the distribution of these fungal producers is shown in Figure 11B. These fungal producers were obtained from a wide range of marine habitats, such as marine sediments (including mudflats and sludges), marine invertebrates (including sponges, soft corals, starfishes, and anemones), and marine plants (algae and mangroves). Marine sediments, marine sponges, marine algae, seawater, soft corals, and mangroves are dominating origins of these fungal strains, with 43, 23, 21, 19, 18, and 8 of the reported compounds characterized (Figure 11B).

**Figure 12:**
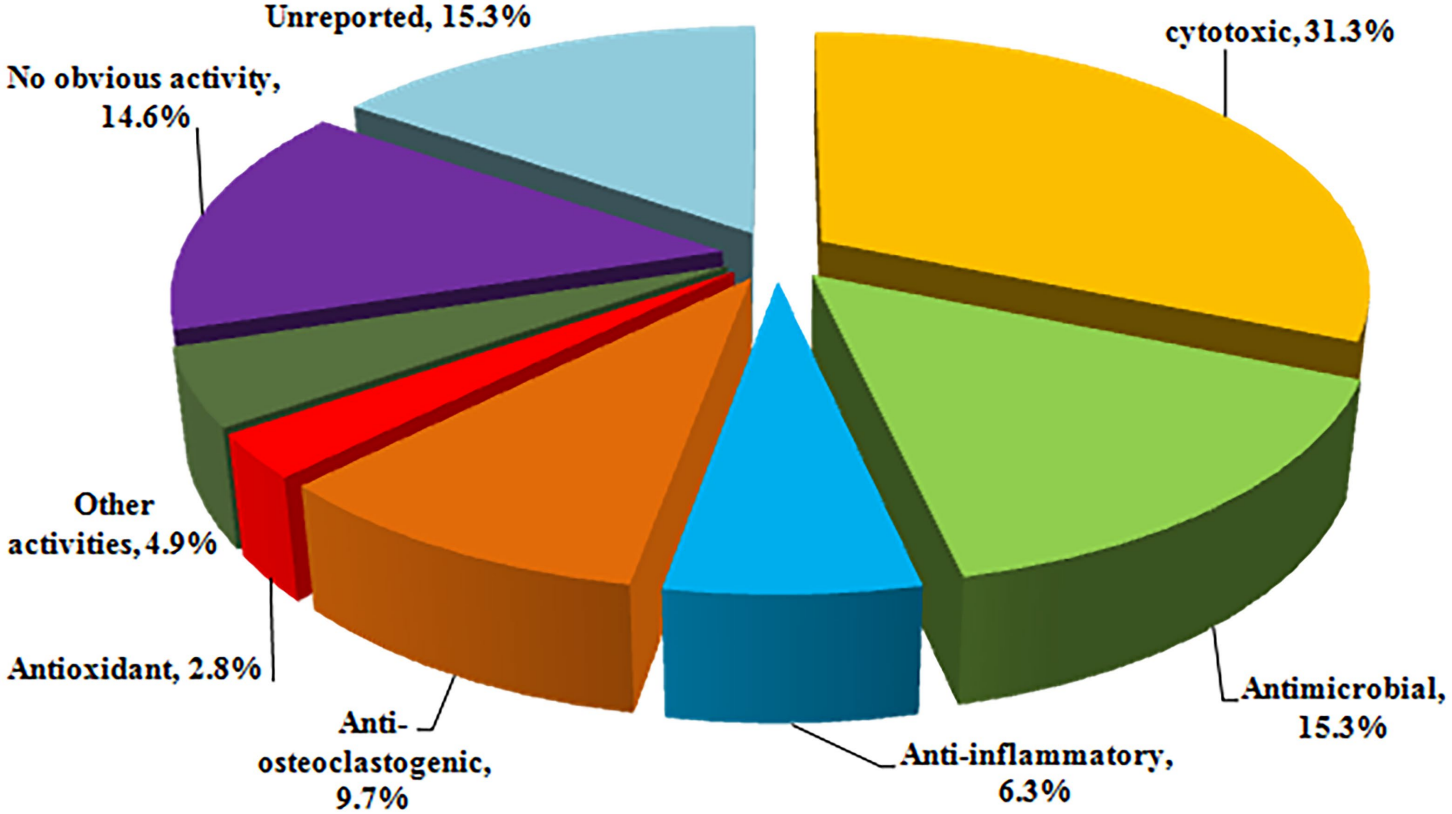
Percentages of bioactivities of halometabolites.

Halometabolites are vital sources for new drugs discovery given to their high diversity in structures and bioactivities. It is considered that the presence of halogen substituents profoundly enhances the bioactivity of natural compounds, as it is obvious that halometabolites often possess higher biological activity than that non-halogen substituted natural compounds. However, it lacks solid evidence that compounds with two or more halogen substituents, such as compounds **25**–**27** with two chlorine groups, **40**–**48** with two chlorine groups, and **134**–**135** with three chlorine groups, exhibit better activity than those with single substituent. The reported halometabolites derived from marine fungi demonstrated pronounced biological activities, including cytotoxic, antimicrobial, anti-inflammatory, antioxidant, and enzyme inhibitory properties (Figure 12). 31.3% of the isolated halometabolites were found to possess certain cytotoxicities. More importantly, some of them showed even higher activity than the positive controls. For example, the chlorinated azaphilones **1**, **2**, and **5** showed significant cytotoxic activity against the human gastric cancer MGC803 and AGS cell lines at a nanomole level (Wang et al., 2020), while compounds **19**, **21** and **22** were found to possess anti-methicillin resistant *S. aureus* activity with MICs of 7.3–7.8 μg/ml (the positive control chloramphenicol, MIC = 7.6 μg/ml) (Wang et al., 2018). The phenalenone **74** displayed higher activity against MRCNS (MIC = 25.0 μM) and MRSA (MIC = 12.5 μM) than the positive control ciprofloxacin (MICs of 25.0 and > 50 μM, respectively), indicating the high potential of these heptaketide phenalenones as lead compounds for drug-resistant pathogens (Han et al., 2021). The iodinated dimeric naphtho-γ-pyrone **76** displayed potent antimicroalgal activity against the marine microalgae *Prorocentrum minimum* with an IC_50_ value of 0.61 μg/ml, compared with the positive control CuSO_4_ (IC_50_ = 2.4 μg/ml) (Li et al., 2022). It is well-known that some halometabolites have been on the market for decades as pharmaceuticals, as exemplified of antibiotic chloramphenicol and pyrrolnitrin and antitumor rebeccamycin. The promising bioactivities indicate that searching for new halometabolites is an important way to develop new drugs and agrochemicals.

In conclusion, in the exploration of bioactive natural compounds, we focus on the potential of marine-derived fungi as producers of halometabolites. This comprehensive review illustrates the chemistry and biological activities of halometabolites produced by marine-derived fungi. 145 halogenated compounds, including 118 chlorinated, 23 brominated, and 4 iodinated, which are classified into polyketides (62.7%), phenols (16.6%), alkaloids (14.5%), and terpenoids (6.2%), were isolated from 17 genera of marine-derived fungi. Their pronounced biological activities, such as cytotoxic, antimicrobial, anti-inflammatory, antioxidant, and enzyme inhibitory properties, revealed a high potential of these halogenated compounds as lead compounds for drug discovery. It should be pointed out that despite a large number of new halometabolites have been characterized; those halogenated compounds are relatively unexplored. Further OSMAC method by changing the cultural conditions will induce the production of more halometabolites.

## Data availability statement

The original contributions presented in the study are included in the article/Supplementary material, further inquiries can be directed to the corresponding authors.

## Author contributions

YC, L-CX, and SL: collected and reorganized the literature data. YC: wrote this manuscript. Z-XZ and G-YC: conceived the ideas and revised this manuscript. All authors contributed to the article and approved the submitted version.

## Conflict of interest

The authors declare that the research was conducted in the absence of any commercial or financial relationships that could be construed as a potential conflict of interest.

## Publisher’s note

All claims expressed in this article are solely those of the authors and do not necessarily represent those of their affiliated organizations, or those of the publisher, the editors and the reviewers. Any product that may be evaluated in this article, or claim that may be made by its manufacturer, is not guaranteed or endorsed by the publisher.
