# Supplementary material for: Halometabolites isolated from the marine-derived fungi with potent pharmacological activities
Source: Front Microbiol. 2022 Oct 4;13:1038487. doi: 10.3389/fmicb.2022.1038487 (PMC9576957; doi:10.3389/fmicb.2022.1038487)
Supplement: Supplementary file 1 [file Table_1.pdf]

Supplementary Material

Halometabolites isolated from the marine-derived fungi with potent pharmacological activities

Yu Chen[1], Lian-Cheng Xu[1], Shan Liu[1], Zi-Xiang Zhang[2*], Guan-Yi Cao[1*]

[1]Department of General Surgery, Suqian First Hospital, Suqian 223800, People's Republic of China

[2]Department of General Surgery, The First Affiliated Hospital of Soochow University, Suzhou 215006, People's Republic of China

***Correspondence:**

Guan-Yi Cao: cheerychy@163.com

Zi-Xiang Zhang: zhangzxz66@163.com

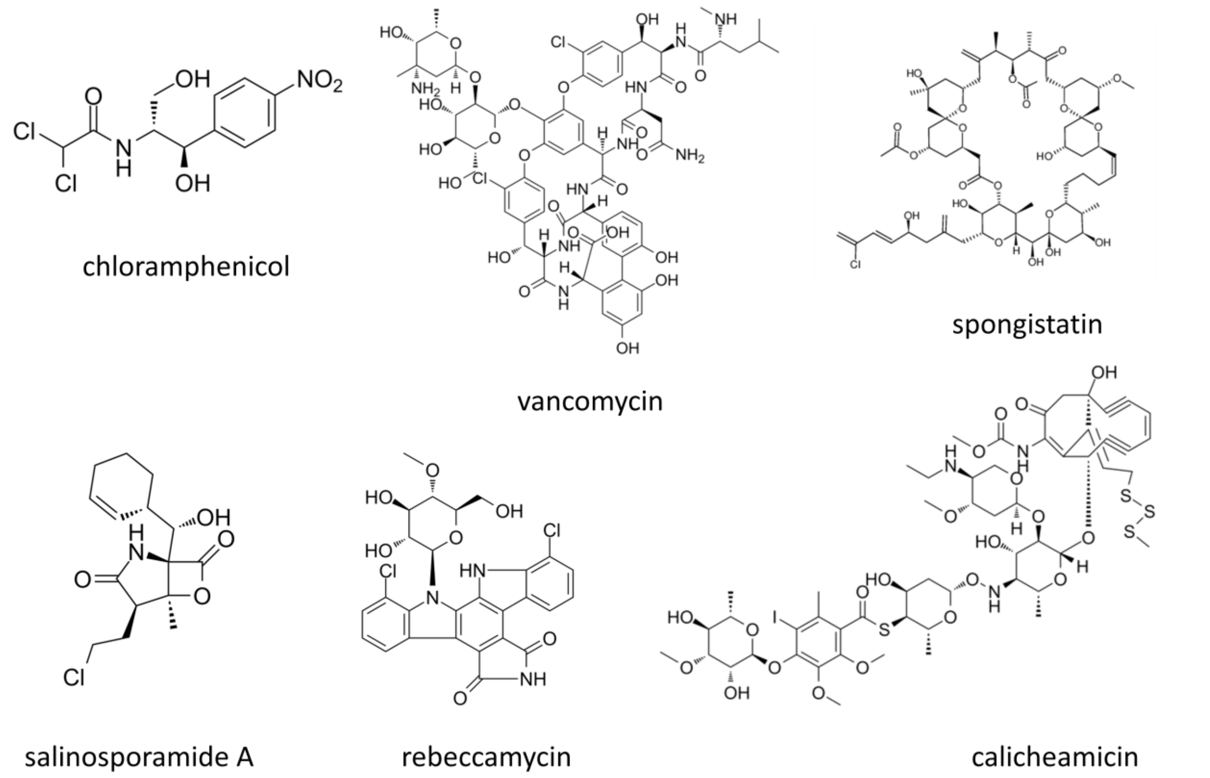

Figure S1 Representative examples of halogenated natural products-inspired pharmaceuticals
